# Enhancing Patient Comprehension in Skull-Base Meningioma Surgery through 3D Volumetric Reconstructions: A Cost-Effective Approach

**DOI:** 10.3390/jpm14090982

**Published:** 2024-09-16

**Authors:** Gheorghe Ungureanu, Larisa-Nicoleta Serban, Lehel Beni, Stefan-Ioan Florian

**Affiliations:** Department of Neurosciences, “Iuliu Hatieganu” University of Medicine and Pharmacy Cluj, 400347 Cluj-Napoca, Romanialehelbeni@yahoo.com (L.B.);

**Keywords:** meningioma, imaging, three-dimensional, cost-benefit analysis

## Abstract

Background: Understanding complex neurosurgical procedures and diseases, such as skull-base meningiomas, is challenging for patients due to the intricate anatomy and the involvement of critical neurovascular structures. Enhanced patient comprehension is crucial for satisfaction and improved clinical outcomes. Patient-specific 3D models have demonstrated benefits in patient education, though they are costly and time-intensive to produce. This study investigates whether the use of 3D volumetric reconstructions with anatomical segmentation, widely available via neuronavigation software, can improve patients’ understanding of skull-base meningiomas, surgical procedures, and potential complications. Materials and Methods: This study included twenty patients with skull-base meningiomas. Three-dimensional volume reconstructions and anatomical segmentations were created using preoperative MRI sequences with neuronavigation software. These reconstructions were used during patient consultations where a surgeon explained key aspects of the disease, the surgical intervention, and potential complications. A questionnaire assessed the patients’ perceptions of the utility of these 3D reconstructions. Results: The majority of patients (75%) found the 3D volumetric reconstructions and anatomical segmentations to be more beneficial than MRI images for understanding their disease. Similarly, 75% reported improved comprehension of the surgical approach, and 85% felt that the reconstructions enhanced their understanding of potential surgical complications. Overall, 65% of patients considered the 3D reconstructions valuable in medical consultations. Conclusions: Our study indicates that using accessible, cost-effective, and non-time-consuming 3D volumetric reconstructions with anatomical segmentation enhances patient understanding of skull-base meningiomas. Further research is necessary to confirm these findings, compare these reconstructions with physical 3D models and virtual reality models, and evaluate their impact on patient anxiety regarding the surgical procedure.

## 1. Introduction

Good communication is one of the pillars of establishing a good patient–doctor relationship [1]. A good understanding of their disease improves both patient outcomes and their satisfaction with their doctor [2]. Understanding the complex anatomy of the cranium, the brain parenchyma, and the neurovascular interactions and how a certain pathology affects these structures requires a significant learning curve [3]. This complicates a patient’s understanding of neurosurgical diseases, as most educational materials are presented at a level beyond the recommended comprehension standard [4].

Imaging technologies such as computed tomography (CT) and magnetic resonance imaging (MRI) have been crucial in diagnosing surgical diseases, particularly in neurosurgery. Additionally, they have become indispensable in communicating diagnosis, treatment, and possible complications, forming a standard component of the initial communication between surgeon and patient. The disadvantage is that they only offer a cross-sectional view, which can be challenging to comprehend [3].

In recent years, integrating 3D models into medicine has mainstreamed, especially in surgical specialties, offering insights into complex anatomical structures and facilitating advanced surgical planning and execution. They can also serve as tools for educating patients regarding their disease [5]. Skull-base meningiomas are one of the most complex pathologies in neurosurgery since the combination of the intricate anatomy in a tight space and the importance of the neurovascular structures associated with the distortion created by the tumor generates the premises for elaborate surgical procedures and 3D models can prove extremely valuable in the setting of this pathology [6]. Studies indicate that the use of patient-specific 3D models can provide a better understanding compared to disease-specific models [7].

A disadvantage of using patient-specific 3D models is that they require equipment—3D printers and usable materials—and dedicated personnel, which can involve significant costs [8].

Neuronavigation hardware and software have been widely adopted, and most neurosurgical institutions possess multiple pieces of such equipment. A notable feature of this technology is its ability to generate 3D volumetric reconstructions effortlessly, enabling straightforward 3D anatomical object segmentations with superior accuracy and ease of use compared to other imaging processing software (Brainlab View 4.0, Munich, Germany). Due to their routine use during surgery, neurosurgeons are highly familiar with utilizing these capabilities.

In this study, we aimed to evaluate whether 3D volumetric reconstructions, employing anatomical segmentation to delineate the interactions between skull-base meningiomas and adjacent normal structures, generated via neuronavigation station software (Navigation Software Version 2.1, CA, USA), enhance patients’ comprehension of their pathology, surgical objectives, and potential complications.

## 2. Materials and Methods

### 2.1. Inclusion Criteria

Our study included twenty patients scheduled for elective surgery for the treatment of skull-base meningioma at our department during the year 2023. These patients were selected if they met the following inclusion criteria: (1) a confirmed diagnosis of skull-base meningioma, as determined by contrast-enhanced magnetic resonance imaging (MRI); and (2) patients in good clinical condition without communication impairments, ensuring that they could fully engage in consultations and participate in the study.

Patients requiring emergency surgery or with significant neurological deficits that impaired normal cognition were excluded from the study. Specifically, individuals whose preoperative neurological examination revealed cognitive dysfunction or severe impairments in communication were excluded.

### 2.2. Three-Dimensional Volumetric Reconstruction with Anatomical Object Segmentation

Upon admission for surgical intervention, all patients underwent a standard imaging protocol with a contrast MRI. These contrast-enhanced MRI scans were further processed to generate 3D volumetric reconstructions. The reconstructions were produced using Brainlab View 4.0 (Brainlab, Munich, Germany), a neuronavigation software that can be used for creating precise 3D models for surgical navigation.

The generation of 3D volumetric reconstructions involved anatomical segmentation, where the boundaries of the skull-base meningioma were outlined in relation to critical neurovascular structures. Although these 3D models were approximate, as they were not intended for direct use during the surgical procedure, they provided visualizations of the tumor’s interactions with surrounding anatomical structures. They included features such as the optic nerve, internal carotid artery, basilar artery, and brainstem, depending on the tumor’s location.

### 2.3. Conducting the Consultation

Following the creation of 3D volumetric reconstructions, each patient participated in a follow-up consultation with a member of the surgical team. A computer equipped with the Brainlab neuronavigation software was installed in a designated consultation room specifically for this purpose. During the session, the patient was seated beside the surgeon to facilitate discussions, and the computer screen was divided into three sections.

One section displayed the axial, coronal, or sagittal views of the contrast-enhanced MRI, providing the patient with a 2D perspective of the tumor. Another section showcased a 3D volumetric reconstruction of the patient’s entire head, which could be rotated, zoomed in, and manipulated in real time to highlight specific anatomical regions. The third section provided a 3D object segmentation that delineated the tumor and its relationship with critical neurovascular structures (e.g., the optic nerve, carotid artery).

During the consultation, the surgeon utilized the 3D reconstructions to explain several key aspects of the surgical process. This included detailing the proposed surgical approach, such as where the incision and craniotomy would be made, and describing the steps involved in tumor resection. The 3D reconstructions were used by the surgeon to convey how the tumor interacted with surrounding neurovascular structures and the specific challenges that might arise during the operation. Potential complications, such as damage to arteries or nerves, were discussed, with the 3D models providing a visual reference to illustrate these risks more clearly. The patients were encouraged to ask any questions they had during the explanation.

### 2.4. Questionnaire

After the consultation, each patient was given a questionnaire in their native language (Romanian) to evaluate the impact of using 3D volumetric reconstructions alongside traditional imaging techniques. The purpose of this questionnaire was to determine whether these visual tools enhanced the patients’ understanding of several areas. These included the clarity of the surgeon’s explanation of their medical condition, the details of the proposed surgical treatment, the tumor’s interaction with nearby neurovascular structures, and the potential risks and complications associated with the surgery. The questionnaire also sought the patients’ feedback on whether they believed 3D reconstructions should be integrated as a standard practice in medical consultations to improve communication between doctors and patients. A detailed English version of the questionnaire can be found in Table 1.

## 3. Results

Patient gender, age, highest level of education, and tumor location are detailed in Table 2.

**Figure 1:**
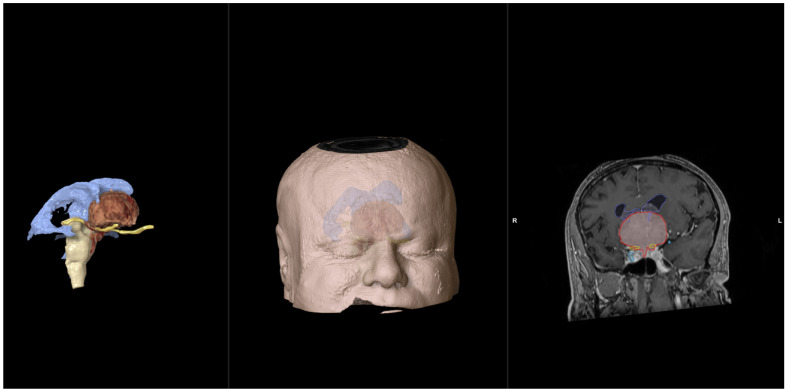
In each instance, the screen was divided into three sections. In this specific case, the patient with a tuberculum sellae meningioma was experiencing visual impairment, predominantly on the right side. The tumor had partially engulfed the right carotid artery. Utilizing the 3D reconstructions of the head, the surgical approach and craniotomy sites were detailed. Anatomical segmentation was employed to explain the necessity of decompressing the optic nerve encroached by the tumor and the potential risk of carotid artery injury (blue: ventricles; white: brainstem; brown: tumor: yellow: optic nerves; red: carotid artery. R; right side).

The questionnaire results are illustrated in Figure 1. A notable finding is that none of the patients selected the first option for any of the six questions, indicating that none perceived the 3D models as negatively impacting their understanding of the disease, its treatment, or their interaction with the physician. Additionally, no patient found the 3D models to be useless when compared to traditional 2D MRI imaging.

The 3D volumetric reconstructions were found to be beneficial across all assessed areas: compared to traditional imaging, in understanding the proposed surgical approach, and in grasping the potential complications associated with surgery. Specifically, 75% of patients felt that the reconstructions were useful in understanding their condition compared to standard MRI images, and the same percentage found them to significantly enhance their understanding of the proposed surgical approach and technique. Notably, 20% of patients (one in five) reported that the reconstructions were extremely helpful in understanding their disease. However, 25% indicated that the 3D reconstructions offered only limited additional benefits compared to traditional MRI imaging.

**Figure 2:**
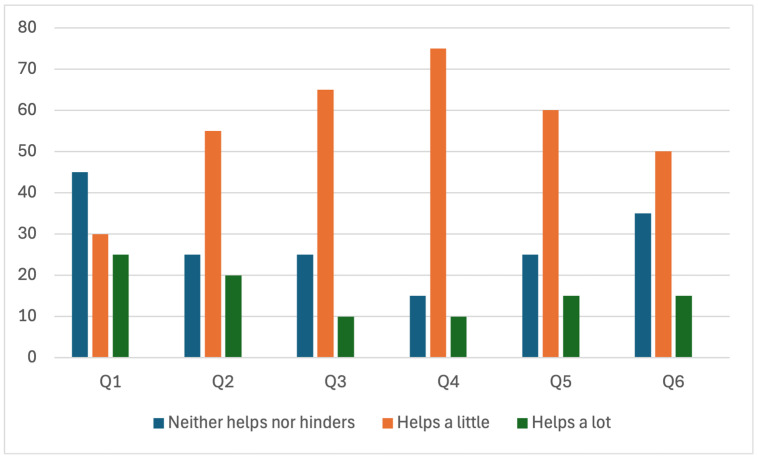
Percentage of patients who selected each option for the given questions. Notably, no patient chose the first option for any of the questions.

The greatest benefit provided by the 3D reconstructions was in helping patients understand the potential complications associated with surgery, with 85% of patients reporting a high or very high benefit. This is particularly relevant, as understanding the relationship between the tumor and nearby neurovascular structures at risk during surgery is often challenging for individuals without extensive medical knowledge. Visual aids significantly improved this comprehension, especially in illustrating the tumor’s proximity to critical structures, such as the carotid artery or optic nerves, which are frequently affected by the tumor. In this context, 75% of patients reported a substantial benefit in understanding the anatomical location of their tumor (Figure 2).

Overall, 65% of patients expressed the belief that 3D volumetric reconstructions should be routinely used during medical consultations.

One in four patients (25%) reported that their understanding of the doctor’s explanations was significantly improved by the 3D volumetric reconstructions, while another 30% found them to be very helpful for their comprehension. However, 45% stated that these reconstructions did not add any value to the doctor’s explanation. This suggests that while the 3D models can be beneficial, the doctor’s explanation remains the primary and most crucial factor in patient understanding.

## 4. Discussion

We conducted a study utilizing 3D volumetric reconstructions with anatomical segmentation, created on a neuronavigation station, to enhance preoperative understanding for patients with skull-base meningiomas. This method, which incurs no significant costs or time requirements, was used to improve patients’ comprehension of their condition, proposed treatment, and potential complications. The reconstructed images facilitated detailed preoperative discussions, clarifying surgical approaches, goals, and possible challenges. Our findings indicate that these personalized reconstructions significantly enhance patient understanding compared to traditional discussions based on standard MRI images. To our knowledge, this is the first study to assess the effectiveness of 3D volumetric reconstructions generated with neuronavigation software for patient education.

### 4.1. The Need to Improve Doctor–Patient Communication in Skull-Base Neurosurgery

Neurosurgery is by far the most exposed medical specialty to malpractice claims. Although worldwide data are lacking, in the US, approximately 20% of neurosurgeons have malpractice claims each year, and virtually all neurosurgeons face a malpractice suit up to the age of 65 [9]. Poor communication between physicians and patients has been quoted repeatedly as the leading cause of malpractice suits, independent of outcomes [10].

Numerous studies indicate that a significant contributor to malpractice claims across various regions globally is the inadequate understanding of medical procedures and their associated risks by patients or their families [4,11]. This issue frequently stems from a lack of comprehensive health education, with most of the research advocating for the simplification of materials used in medical education [12]. There is a difference in the degree of patients’ understanding depending on clinical settings and the pathology [13,14]. Nonetheless, health literacy, or one’s ability to obtain and understand basic health information, is generally low, especially in neurosurgery [15]. As skull-base procedures carry with them high risks and are prone to complications, good surgeon–patient communication, including a good understanding of the patient regarding the aims of the surgical procedure and potential pitfalls, is essential.

### 4.2. The Importance of Using Visual Aids to Enhance Patients’ Understanding of Their Condition

Multiple studies have shown that written material regarding neurosurgical procedures is written above the recommended level for a patient without a medical background to have a good understanding independent of the source [4]. “A picture is worth a thousand words” certainly holds true in medicine. However, understanding CT and MRI imaging can be challenging for patients, as it requires a certain level of familiarity with underlying anatomical structures [16,17]. Numerous studies have demonstrated that the use of 3D models and reconstructions significantly enhances patients’ comprehension of their disease, the necessary treatment, and the potential complications associated with surgery [5,7,17]. Our study supports this hypothesis. Given that improved patient understanding can lead to increased satisfaction with treatment, thereby strengthening the surgeon–patient relationship, the use of 3D models may also serve as a valuable tool in reducing patient dissatisfaction with medical procedures.

By providing clear, visual representations of complex anatomical structures, such as 3D volumetric reconstructions, patients can better grasp the nature and extent of their condition. A total of 75% of the patients in our study found these reconstructions to be beneficial when compared to traditional MRI. Visual aids bridge the gap between medical knowledge and patient comprehension, making it easier for individuals to visualize the relationship between their disease and surrounding structures, understand proposed treatments, and appreciate potential risks and complications. This improved understanding not only empowers patients to make informed decisions about their care but also fosters more effective communication between physicians and patients.

### 4.3. The Significance of Strong Communication Skills in Enhancing the Doctor–Patient Relationship and Improving Patient Mental Health

It is important to recognize that no tool can compensate for poor communication from the surgeon. In our study, nearly half of the patients felt that 3D reconstructions neither hindered nor enhanced the doctor’s explanations. This strongly suggests that the most valuable asset a surgeon has for improving patient compliance and fostering a strong doctor–patient relationship is effective communication skills. While adjuncts such as MRI images and 3D reconstructions can aid in patient understanding, they cannot replace or make up for inadequate communication, as highlighted by numerous studies [4,10,18].

While our study did not address this question specifically, other studies have demonstrated that adequate information regarding diagnosis and treatment is positively associated with improved mental health outcomes, and there is a notable correlation between dissatisfaction with the information provided and the development of depression in patients with neoplastic diseases [19,20]. For example, a study involving neurosurgical patients diagnosed with high-grade gliomas found that enhanced communication about the disease and the required treatments led to reduced levels of anxiety [13]. As previously noted, our study appears to align with findings from other research, which suggest that the use of 3D models enhances patient understanding. The literature further supports the notion that this increased understanding can subsequently reduce the patient’s anxiety related to the medical procedure. Although we did not utilize specific instruments to measure anxiety levels in our study, we hypothesize that 3D volumetric reconstructions could be valuable tools in alleviating anxiety concerning surgical procedures.

### 4.4. Technical and Financial Aspects of Using 3D Anatomical Models in Low-Resource Clinical Settings

Research indicates that patient-specific models significantly enhance patient understanding compared to non-specific models [16,17]. Additionally, 3D models, both printed and virtual reality-based, are increasingly being utilized for the education of medical professionals at all levels, from students to attending physicians, and are already in use for this purpose in various settings [6,8,21,22]. While some medical institutions have created 3D printing facilities, this is still unaffordable in most settings worldwide, as the costs are relatively high [3,21]. Most equipment needs some engineering background for computer-assisted design (CAD) techniques and troubleshooting [8]. The production of these models also takes several days, possibly driving up the expenses associated with the medical management of the patients [21]. The use of virtual reality (VR) 3D techniques can provide better interaction with the models and no printing expenses. Still, the costs of acquiring specialized software and hardware are barriers to access in medical settings with limited resources [21,22].

In contrast, neuronavigation is widely utilized in neurosurgery and is regularly applied across a variety of pathologies to guide the surgeon. As technological costs continue to decrease with broader adoption, neuronavigation is likely to become increasingly accessible, even in neurosurgical institutions with limited resources. Given that most neuronavigation software from different companies can generate 3D reconstructions, we propose that the use of 3D volumetric reconstructions presents a cost-effective method to enhance education, benefiting not only patients but also students, residents, and attending physicians from low-resource settings.

All the 3D volume reconstructions in our study were performed after the patient was admitted to the hospital. They did not involve any extra costs or delay until surgery. Since neuronavigation is usually employed during surgical interventions, the only extra step not usually performed in our institution is the underlining of the tumor and vessels using a special tool of the neuronavigation software. The software we used has the capacity to recognize normal brain tissue, optic nerves, and ventricles on a standard MRI with enough images, and recalibration of these structures requires minimal effort. We did not attempt to create highly detailed reconstructions that precisely replicate the local anatomy, as the generated reconstructions were not intended for use during the surgical procedure.

Given that some surgeons may prefer to use detailed 3D neuronavigation-generated volume reconstructions during surgery, and considering that most contemporary systems allow for the export of these 3D models, creating accurate models for various pathologies can also be beneficial in outpatient settings during the initial consultation between doctor and patient. These reconstructions are cost-effective to produce and export, and they can be easily viewed on any computer. When patients are accompanied by their relatives, these models become even more valuable. They provide patients and their families with a clearer understanding of the condition and treatment options, allowing ample time for the patient to discuss their preferences with loved ones and to formulate questions they may wish to address with the surgeon.

### 4.5. Limitations of This Study

This study has several limitations. The studied population was small, which did not allow us to see if the educational background of the patients or the grade of literacy influenced the degree of understanding. No follow-up was conducted, so the long-term effects of improved preoperative understanding on patient satisfaction cannot be determined from our study. Finally, we did not conduct any test to assess the influence of the 3D presentation on the patients’ anxiety levels regarding the procedure. Future tests need to assess whether this method of interaction leads to a better understanding in all cases, if the educational background of the patients limits it, and whether it influences the anxiety level surrounding the surgical procedure.

## 5. Conclusions

Individualized 3D patient-specific models can improve patient education about their disease, but their creation is time-consuming and resource-intensive. We demonstrate that utilizing affordable, highly available individualized 3D volume reconstructions with anatomic object segmentation generated on neuronavigation software can improve patient comprehension of their condition, the surgical approach, lesion resection, and potential complications in cases of skull-base meningiomas. These reconstructions can enhance the efficiency of surgeon–patient communication, potentially improving long-term outcomes and reducing patient dissatisfaction. Further studies are needed to compare these reconstructions to physical 3D models and to assess their impact on anxiety levels related to the surgical procedure.

## Figures and Tables

**Table 1:** Questionnaire to assess the effectiveness of 3D reconstructions.

I believe that the 3D reconstruction helped me understand the doctor’s explanations:	Less	Neither helps nor hinders	Helps a little	Helps a lot
Compared to MRI images, I believe the 3D reconstruction helped me understand my disease:	Less	Neither helps nor hinders	Helps a little	Helps a lot
I believe the reconstruction helped me understand the proposed surgical approach and technique:	Less	Neither helps nor hinders	Helps a little	Helps a lot
I believe the 3D reconstruction helped me understand possible surgical complications:	Less	Neither helps nor hinders	Helps a little	Helps a lot
I believe the 3D reconstruction helped me understand better where the tumor is located and its relationship with other structures:	Less	Neither helps nor hinders	Helps a little	Helps a lot
I believe 3D reconstructions should be used in medical consultations:	Less	Neither helps nor hinders	Helps a little	Helps a lot

**Table 2:** Patient characteristics: gender, age, level of education, tumor location.

Gender	Age	Level of Education	Tumor Location
Female	55	high school	Petroclival
Female	53	college	Tuberculum sellae
Female	60	college	Clinoid process
Female	64	high school	Clinoid process
Female	65	high school	Clinoid process
Female	53	high school	Tuberculum sellae
Female	46	high school	Petroclival
Female	29	high school	Clinoid process
Female	48	college	Tuberculum sellae
Female	55	high school	Tuberculum sellae
Female	61	high school	Tuberculum sellae
Male	68	technical school	Petroclival
Female	63	technical school	Clinoid process
Female	64	high school	Clinoid process
Female	66	college	Tuberculum sellae
Male	55	technical school	Clinoid process
Female	64	high school	Tuberculum sellae
Female	47	college	Clinoid process
Male	67	technical school	Tuberculum sellae
Female	55	high school	Tuberculum sellae

## Data Availability

The data presented in this study are available on request from the corresponding author due to the fact that they include patient personal data.
